# Effect of physical interventions on physical performance and physical activity in older patients during hospitalization: a systematic review

**DOI:** 10.1186/s12877-018-0965-2

**Published:** 2018-11-23

**Authors:** Kira Scheerman, Kirsten Raaijmakers, René Hubert Joseph Otten, Carel Gerardus Maria Meskers, Andrea Britta Maier

**Affiliations:** 1 Department of Internal Medicine, Section of Gerontology and Geriatrics, VU University Medical Center, De Boelelaan 1117, 1081 HV Amsterdam, The Netherlands; 2 Department of Human Movement Sciences, @AgeAmsterdam, Faculty of Behavioural and Movement Sciences, Amsterdam Movement Sciences, Van der Boechorststraat 7, 1081 BT Amsterdam, The Netherlands; 3 Medical Library, Vrije Universiteit Amsterdam, De Boelelaan 1117, 1081 HV Amsterdam, The Netherlands; 4 Department of Rehabilitation Medicine, VU University Medical Center, De Boelelaan 1117, 1081 HV Amsterdam, The Netherlands; 5 Department of Human Movement Sciences, VU University, Amsterdam, the Netherlands, De Boelelaan 1105, 1081 HV Amsterdam, The Netherlands; 6 Department of Medicine and Aged Care, @AgeMelbourne, Royal Melbourne Hospital, University of Melbourne, Melbourne, Australia, Clinical Sciences Building, Royal Parade, Parkville, VIC 3010 Australia

**Keywords:** Exercise, Physical therapists, Aged, Hospitalization

## Abstract

**Background:**

To counteract decline in physical performance and physical activity in older patients during hospitalization, multiple physical interventions were developed. However, it is unknown whether these are effective in this particular population. This systematic review aimed to identify the effect of physical interventions on physical performance and physical activity in older patients during hospitalization.

**Methods:**

The systematic search included PubMed, EMBASE, Cinahl, the Trials database of The Cochrane Library and SPORTdiscus from inception to 22 November 2017. Studies were included if the mean age of the patient cohort was 65 years and older and the effect of physical interventions on physical performance or physical activity was evaluated during hospitalization.

**Results:**

Fifteen randomized controlled trials met the inclusion criteria. Overall, the effect of physical interventions on physical performance was inconsistent. Patient tailored interventions, i.e. continuously adapted to the capabilities of the patient were not found to be superior over interventions that were not. Physical activity as outcome measure was not addressed. Reporting of intensity of the interventions and adherence were frequently lacking.

**Conclusions:**

Evidence for the effect of physical interventions on physical performance in older patients during hospitalization was found uncertain. Further research on the efficacy of the intervention is needed, comparing types of intervention with detailed reporting of frequency, intensity and duration.

**Electronic supplementary material:**

The online version of this article (10.1186/s12877-018-0965-2) contains supplementary material, which is available to authorized users.

## Background

Older age is associated with a high prevalence of age related diseases and is a major risk factor to become admitted to hospitals [1]. Hospitalization is associated with a decline in physical performance [2], nursing home admission [3] and short term mortality [4]. A decline in physical performance is likely to be aggravated by low physical activity during hospitalization [5], which in turn affects activities of daily living [6] and occurrence of falls [7]. Older patients have decreased physiological and functional reserves that renders them vulnerable to negative effects of low physical activity during hospitalization [8].

In recent years attention has been paid to improving hospital outcomes especially for the vulnerable group of older patients [9]. Multiple physical interventions have been developed to enhance physical performance and physical activity in hospitalized patients. Physical interventions include examples such as exercise prescribed by health care professionals, supervised exercise sessions and physician counseling during hospitalization [10]. A meta-analysis showed that physical therapy of higher intensity, i.e. longer duration and higher frequency of sessions, reduce length of hospital stay and improve physical performance in patients older than 18 years with sub-acute and acute conditions [11]. In older hospitalized patients positive effects of multidisciplinary programs with an exercise component have been reported, however, the effects of solely a physical intervention were inconclusive [10, 12]. Principal elements of these programs included goal setting tailored to the individual patient and interventions tailored to the patients’ needs [13]. To improve patient outcome it is important to identify what type of physical interventions positively affects physical performance and physical activity in older patients during hospitalization.

This systematic review aimed to identify the effect of physical interventions on physical performance and physical activity in older patients during hospitalization. Additionally, we aimed to compare the effect of patient tailored physical interventions e.g. continuously adapted to the capabilities of the patient to the effect of non-patient tailored interventions. We hypothesize that physical interventions improve physical performance and physical activity and that physical interventions continuously adapted to the capabilities of the patient are superior over interventions that are not.

## Methods

### Search strategy

For this systematic review a literature search was performed by RO and KR. To identify relevant publications about physical interventions of hospitalized patients with an age of 65 years or more we performed searches in the bibliographic databases PubMed, EMBASE.com, Cinahl (via Ebsco), the Trials database of The Cochrane Library (via Wiley) and SPORTdiscus (via Ebsco) from inception to 2017 November 22nd. Search terms included controlled terms (as MeSH in PubMed and Emtree in Embase etc.) as well as free text terms. We used free text terms only in The Cochrane Library. Search terms expressing the age of the patients were used in combination with search terms for physical interventions and search terms for hospitalization. The search was then limited to randomized controlled trials. The full search strategies for all databases can be found in Additional file 1.

### Study selection

Studies were assessed for eligibility by screening title and abstract by two researchers (KR and KS) for the following inclusion criteria: the study population or sub group consisted of hospitalized older patients with a mean age of 65 years and older, and the studies contained physical interventions during hospitalization with physical performance or physical activity as outcome measures. Physical performance was defined as the ability to perform a physical task at a desired level. Physical activity was defined as any bodily movement produced by skeletal muscles requiring energy expenditure. Studies were excluded when: other study designs than randomized controlled trials (RCTs) were used, pre and post measurements were not performed during hospitalization, articles were written in other languages than English or Dutch and physical interventions were performed to improve disease related outcomes of patients affected by Chronic Obstructive Pulmonary Disease (COPD), chronic heart failure, stroke, hip fracture or knee replacement. These patient groups were excluded due to the disease specific interventions. In case of uncertainty for inclusion the articles were discussed with a third researcher (CM/AM).

### Data extraction and analysis

Data extraction was completed by two researchers (KS and KR). Data extracted of each study included reason for hospitalization, setting, patient group characteristics (number and age of patients of intervention and control group), intervention characteristics (type of intervention, patient tailored, frequency, intensity and duration of the intervention), adherence, moment of pre and post measurement, outcomes measures (primary and secondary), change in physical performance and physical activity between pre and post measurement was extracted and PEDro score [14]. Interventions were considered patient tailored if the intervention was adapted to the capabilities of the patients prior to and during the intervention. Frequency was defined as the number of intended physical intervention sessions per week and intensity as the number of repetitions or level of exertion and duration of one session. Duration of a session was expressed as the number of minutes of one session and duration of the intervention as the number of days the intervention was performed. Adherence was defined as the percentage of the sessions the patient participated against the number of intended sessions during the intervention period.

The extracted data were structured in tables stratified by study characteristics, characteristics of the physical interventions and study outcomes. Conclusions were based on the significance level of the primary outcome and on the number of outcomes with a significant effect. *P*-values equal or lower than 0.05 were considered statistically significant. Studies were grouped by patient tailored and non-patient tailored interventions. Due to the heterogeneity of the interventions and outcomes grouping by type of intervention or outcomes measures was not appropriate. Level of evidence was based on the outcome of the quality assessment of studies and on population size.

## Results

**Fig. 1:**
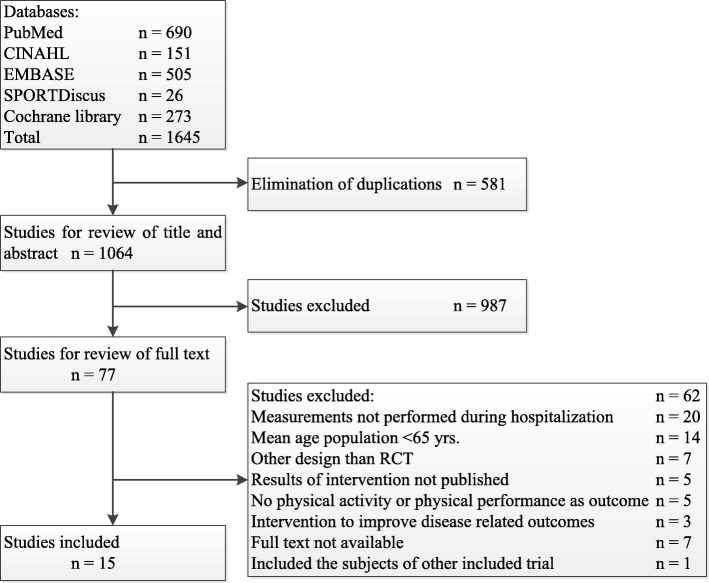
Flowchart of article selection

A total of 1645 studies including 581 duplicates were found. After screening titles, abstracts, and full text 1049 studies were excluded, resulting in 15 studies being included in this systematic review. The study selection process and reasons for exclusion are presented in Figure 1. Overall, type of intervention and outcome measures for physical performance varied widely. None of the studies measured physical activity as outcome. The number of the patients included in the RCTs was low, only one study included more than 200 patients in the intervention group. The study characteristics are presented in Table 1.

**Table 1:** Study characteristics

Author	Year^a^	Reason for hospitalization Setting (acute/ subacute, ward)	Group		Moment of measurement	Outcomes^c^#	PEDro scale^d^
			Intervention N^b^, type, age (y)	Control N^b^, type, age (y)			
Bürge [21]	2017	Dementia Acute, psychogeriatric ward	78, Physical exercise program, 81.7 (7.7)	82, Usual care, 81.1 (7.7)	A, 4 weeks after A	**BI** ^**e**^ **, FIM** ^**f**^	Sufficient
Czyzewski [17]	2013	Major abdominal surgery Subacute, general and colorectal surgery ward	18, Physiotherapy based on elements of proprioceptive neuromuscular facilitation, 75 (5.8)	16, Usual care, 76 (5.6)	3 days before surgery, 4 days after surgery	**10MWT** ^**g**^ **, TUG** ^**h**^ **, SAP** ^**i**^	Sufficient
Haines [23]	2007	Various diagnoses (e.g. orthopaedic, geriatric management, stroke) Subacute, NG	93, Additional exercise program, 83 (IQR 77,88)	80, Usual care, 81 (IQR 75,86)	A, D	**Number of falls**, FRT^j^, TUG^h^, 6MWT^k^, Gait velocity, Step length, Muscle strength	Good
Hegerova [22]	2014	Various diagnoses (e.g. cardiovascular, infection, kidney) Acute, internal medicine ward	100, Rehabilitation and nutritional intervention, 83.6 (3.8)	100, Usual care, 83.2 (3.8)	Day 2 of A, D	**Lean body Mass**, BI^e^,	Good
Jones [25]	2006	NG Acute, general ward	80, Exercise program, 81.9 (8.0)	80, Usual care, 82.9 (7.6)	Within 2 days of A, D	**ModifiedBI** ^**e**^ **, TUG** ^**h**^	Sufficient
Kim [15]	2013	NG Subacute, NG	15, Horse riding simulation, 78.4 (6.2)	15, Ball exercise, 78.5 (6.6)	A, 8 weeks after A	**Romberg test, FRT** ^**j**^ **TUG** ^**h**^ **, 10MWT** ^**g**^	Good
Laver [24]	2012	Various diagnoses (e.g. medical, pain, fall or fracture) Subacute, geriatric rehabilitation ward	22, Interactive gaming program, 85.2 (4.7)	22, Usual care, 84.6 (4.4)	Day 2 of A, D	**TUG**^**h**^**,** ModifiedBBS^l^, SPPB^m^, IADL^n^, FIM^f^, ABC^o^	Good
Maggioni [37]	2009	Various diagnoses (e.g. cardiovascular, orthopaedic, neurological) Subacute, rehabilitation ward	1. 10, Kinesiotherapy (KT), 81.2 (5.9) 2. 10, Electrical stimulation (ES), 84.1 (3.4) 3. 10, KT + ES, 82.2 (7.4)	10, Usual care, 82.1 (5.4)	A, D	**Muscle strength, 6MWT**^**k**^, **Tinetti balance and gait test**	Good
de Morton [19]	2007	Various diagnoses (e.g. respiratory, circulatory, digestive) Acute, medical ward	110, Exercise program, 80 (8.0)	126, Usual care, 78 (7.0)	A, D	**Discharge destination, TUG**^**h**^, BI^e^, FAC^p^,	Good
Oesch [28]	2017	Musculoskeletal impairment Subacute, geriatric rehabilitation ward	26, Self-regulated exergames, 73.8 (IQR 67.9, 79.1)	28, Self-regulated conventional exercises, 74.3 (IQR 66.1, 79.3)	A, 10 days after A	**Adherence,** objective dynamic balance	Good
Parsons [16]	2016	Various diagnoses (e.g. cardiovascular, musculoskeletal, neurological) Subacute, rehabilitation ward	26, Physical therapy and vibration training, 82.1 (6.4)	24, Usual care, 81.8 (8.0)	A, D	**PPA**^**q**^**(muscle strength),** FIM^f^	Good
Raymond [26]	2017	Various diagnoses (e.g. fracture, fall, respiratory) Subacute, rehabilitation ward	231, High-intensity functional exercise, 84.5 (7.3)	223, Usual care, 84.1 (6.9)	A, < 48 before D	**EMS**^**r**^**,** BBS^l^, gait speed, TUG^h^	Good
Said [20]	2012	Various diagnoses (e.g. musculoskeletal, cardiovascular, falls) Subacute, rehabilitation ward	22, Exercise program, 80.8 (4.6)	25, Usual care, 81.6 (6.5)	Within 2 days of A, < 48 h before D	**DEMMI**^**s**^**, EMS**^**r**^**, TUG**^**h**^**,** BI^e^	Good
Tibaek [27]	2013	Various diagnoses (e.g. falls, respiratory, medicine) Subacute, geriatric rehabilitation ward	29, Progressive resistance strength training, 80 (6.5)	27, Usual care, 79 (7.5)	Within 3 days of A, D	**TUG**^**h**^**, 30s-chair stand test, 10MWT**^**g**^**,** BI^e^**,** Modified FAC^p^	Good
Wnuk [18]	2016	Abdominal aortic aneurysm surgery Subacute, general and vascular surgery ward	1. 15, Backward walking training, 68 (3) 2. 16, Forward walking training, 70 (3)	16, Usual care, 69 (4)	A, 7 days after surgery	**6MWT** ^**k**^	Good

All variables are presented as mean (SD) unless indicated otherwise. *NG* Not given, *A* Hospital admission, *D* Hospital discharge, #primary outcomes in bold, *IQR* Interquartile range, ^a^ = Year of publication, ^b^ = Number of patients at baseline, ^c^ = Secondary outcomes included of relevance of this systematic review, ^d^ = PEDro scale: 0–3 = insufficient, 4–5 = sufficient, 6–8 = good, 9–10 = excellent, ^e^ = Barthel Index, ^f^ = Functional Independence Measure, ^g^ = 10-Meter Walk Test, ^h^ = Time Up and Go, ^i^ = Scale of independent postoperative patient’s activity, ^j^ = Functional Reach Test, ^k^ = 6-min Walk Test, ^l^ = Berg Balance Scale, ^m^ = Short Physical Performance Battery, ^n^ = Instrumental Activities of Daily Living Scale, ^o^ = Activities-patient tailored Balance Confidence scale, ^p^ = Functional Ambulation Categories, ^q^ = Physiological Profile Assessment, ^r^ = Elderly Mobility Scale, ^s^ = de Morton Mobility Index

### Characteristics of the physical interventions

The interventions consisted of horse riding simulation, physiotherapy in combination with whole body vibration training, physiotherapy based on elements of proprioceptive neuromuscular facilitation, a physiotherapy program with a backward or forward walking interval training cycle, exercise programs, a rehabilitation and nutritional intervention, interactive gaming program, progressive resistance strength training, electrical quadriceps stimulation or kinesiotherapy (or a combination of both) and exergames on balance, leg strength and flexibility. A description and the characteristics of the interventions are presented in Table 2. The median number of patients in the intervention groups was 30 (15–231). In 13 studies, control groups received usual care, one control group received ball exercise and one control group received self-regulated conventional exercises. All studies were assessed as sufficient or good quality RCTs as defined by PEDro score of 4 or higher (Additional file 2: Table S1).

**Table 2:** Characteristics of the physical interventions

Author	Year^a^	Intervention	Characteristics of the intervention				
			Patient tailored (yes, intermediate, no^b^ and a brief description)	Frequency per week	Duration of one session (min)	Duration of intervention (days)	Adherence (%)^c^
Bürge [21]	2017	Physical exercise program: group training including strength, flexibility, walking and balance.	Yes, intensity of the exercises increased gradually during the training and was adapted to individual patient abilities.	5	30	20	66
Czyzewski [17]	2013	Physiotherapy based on elements of proprioceptive neuromuscular facilitation: respiratory exercises and change of position using manual resistance on sternum, upper and lower limbs, repeated initial stretch and bilateral symmetric moving standards of shoulder girdle and upper limbs.	Yes, exercises were individualized with an intensity in the range of 40–50% of maximal frequency of heart rate, and instructions for individual practice were provided.	5	30	7	NG
Haines [23]	2007	Additional exercise program: by applying therapeutic principles of tai chi with functional movements an activity visualization.	Intermediate, exercises could be tailored to match individual patient abilities.	3	45	27.9	75
Hegerova [22]	2014	Rehabilitation and nutritional intervention: including training of the lower limbs and therapeutic physical training.	Yes, training of lower limbs and therapeutic physical training were tailored to individual patient abilities. Intensity was determined by an increase of heart rate by a maximum of 15 beats. The heart rate was continuously monitored.	24	5 and 15	11 (7)	NG
Jones [25]	2006	Exercise program: for the upper limb, lower limb, and trunk including four levels: 1. bed exercise; 2. sitting exercise; 3. standing/walking exercise; 4. stair exercise.	Intermediate, level of an exercise program was dependent of baseline functional status of the patient. The exercise program was tailored to the individual patient abilities.	14	NG	Median (IQR) 9 (4, 16)	NG
Kim [15]	2013	Horse riding simulation: imitation of three-dimensional movements (forward and backward, left and right, and up and down) of a live horse.	Yes, the speed of the simulator was adjusted to individual patient abilities while the simulator was moving.	5	20	56	NG
Laver [24]	2012	Interactive gaming program: Nintendo Wii Fit activities on balance, strength or developing aerobic capacity.	Intermediate, activities were selected based on individual patient abilities and treatment needs.	5	25	12.3 (5.6)	90
Maggioni [37]	2009	Three lower limb rehabilitation programs:1. Kinesiotherapy (KT); 2. Electrical stimulation (ES); 3. KT + ES	Intermediate, for KT, load of isotonic exercises was adjusted based on the ability to perform a series of 15–20 repetitions and contractions of isometric exercises were kept for 6–10 s for 10–15 repetitions. For ES, stimulation amplitude was set to the patient’s point of discomfort. Stimulation frequency increased from 35 Hz to 75 Hz to 85 Hz per six sessions.	3	45	42	NG
de Morton [19]	2007	Exercise program: for the upper limb, lower limb, and trunk including four levels. 1. bed exercise; 2. sitting exercise; 3. standing exercise; 4. stair exercise.	Yes, level of an exercise program was prescribed by the project physiotherapist and exercises were tailored to the individual patient abilities. Exercise resistance was increased when patients could do 10 repetitions.	10	20–30	Median (IQR) 5 (3.0, 9.8)	NG
Oesch [28]	2017	Exergames: Seven mini-games for balance, leg strength and flexibility including three levels: 1. sitting exercise, 2. standing exercise 3. walking exercise.	Intermediate, exercise level could be tailored to match individual patient balance abilities.	10	60	10	58
Parsons [16]	2016	Physiotherapy and vibration training: group based physiotherapy and individualized progressive walking programs and whole body vibration consisting of six static exercises targeting lower limb muscles.	Yes, load of the vibrating platform was set at 30–50 Hz and the amplitude was adjusted to individual patient abilities so that the heart rate remained below 85% age-predicted maximal heart rate. Volume and intensity increased progressively according to the overload principle.	5 and 3	30–45 and NG	8.8	NG
Raymond [26]	2017	Progressive resistance strength training, exercises lower limb in supported and unsupported positions, and balance exercises challenging postural stability.	Intermediate, exercises targeted varying levels of mobility. Average intensity level was rated by a staff member after each training.	5	45–60	Median (IQR) 12.3 (11.0, 13.5)	NG
Said [20]	2012	Exercise program: for the upper limb, lower limb, and trunk to improve lower limb strength and balance including four levels. 1. bed exercise; 2: sitting exercise; 3: standing / walking exercise; 4: stair exercise.	Yes, exercises were tailored to the individual patient abilities. Progress was monitored during each session and the intervention was modified based on improvements in the patient’s function.	5–10	NG	Median (IQR) 15 (11.5, 20)	90
Tibaek [27]	2013	Progressive resistance strength training: 1.Exercises in sitting position; 2. Stand up from sitting to standing position; 3.Walking sideways; 4. Elevation up and down on the toes performed in standing position; 5. Training in stair-stepping machine.	Intermediate, load of exercises was determined based on 60–70% of one repetition maximum. Load was increased by a 0.5 kg sandbag and by an elastic band with different resistance when the patient reported that the load was easy or moderate on the modified Borg Scale and could do more than 15 repetitions.	4	50	28 (15)	62.5
Wnuk [18]	2016	Physiotherapy program: consisted of education, active exercises of the upper and lower extremities and backward or forward walking on an interval training cycle.	Yes, intensity of the interval training cycle was adjusted to individual patient abilities based on a stress test and a calculated training heart rate. The heart rate and blood pressure were continuously monitored. Workload increased gradually during the training.	18	1–24	7	NG

All variables are presented as mean (SD) unless indicated otherwise. *NG* Not given, ^a^ = Year of publication, ^b^yes = intervention was adapted to the capabilities of the patient prior to and during the intervention, intermediate = intervention was only adapted to the capabilities of the patient prior to the intervention, no = intervention was not adapted to the capabilities of the patient, ^c^ = percentage of sessions the patient participated against the number of intended sessions during the intervention period

All physical interventions were adapted to the capabilities of the patient prior to the intervention; in eight studies the interventions were adapted both prior to and during the intervention. Frequency of the interventions varied between three to 24 sessions per week. Duration of one session varied between one to 60 min and duration of the intervention between five to 56 days. Intensity was only specified in seven studies and adherence in five studies.

### Effects of the physical interventions

Table 3 presents the effect of physical interventions on physical performance and physical activity in older patients during hospitalization. Four of the eight physical interventions that were continuously adapted to the patient’s capabilities showed positive results on physical performance. Horse riding simulation showed a significantly improved gait ability, measured by Time Up and Go (TUG) and 10-Meter Walk Test (10MWT), and balance in the intervention group compared with the control group [15]. Physiotherapy in combination with whole body vibration training showed positive results on Functional Independence Measures (FIM), however, no positive effect was found on muscle strength [16]. Physiotherapy based on elements of proprioceptive neuromuscular facilitation showed a positive result on return of functional independence of basic movement activities after surgery measured by the scale of independent postoperative patient’s activity. No positive effect was found on gait ability, measured by TUG and 10MWT [17]. A physiotherapy program with a backward walking interval training cycle had a positive effect on walking distance after surgery measured with the 6-min Walk Test (6MWT), however, the group with a forward walking interval training cycle showed no positive effect on walking distance compared with the control group [18]. Exercise programs for upper limb, lower limb and trunk and continuously adapted to the patient’s capabilities, had no positive effect on functional independence reflected by Barthel Index (BI) [19, 20]. The exercise program had no effect on recovery from baseline in functional independence [19]. In addition, an exercise program which included group training on strength, flexibility, walking and balance did not show positive results on functional independence measured by BI en FIM [21]. A rehabilitation intervention in combination with nutritional supplementation had no effect on physical performance assessed by BI [22].

**Table 3:** Change in physical performance and physical activity in older patients during hospitalization

Author	Year^a^	Outcome^b#^	Group							P^e^
			Intervention					Control		
			Pre		Δ^cd^			Pre	Δ^cd^	
Bürge [21]	2017	**Barthel Index**	13.4 (4.4)		0 (6.0)			13.3 (4.1)	−0.8 (6.4)	–
		**Functional Independence Measure**	79.7 (21.5)		−0.7 (30.7)			77.7 (19.3)	−3.3 (29.6)	–
Czyzewski [17]	2013	**10-Meter Walk Test, sec**	NG		4.2			NG	4.6	–
		**Time Up and Go, sec**	NG		2.8			NG	3.7	–
		**Scale of independent postoperative patient’s activity**	NG		36.9 (6.9)^f^			NG	28.3 (6.7)^f^	+++
Haines [23]	2011	**Falls, falls/1000 patient-days**	NG		10.0 - pre			NG	21.2 - pre	+++
		Absolute improvement FRT^g^, cm, median, IQR	10 (6, 14.8)		9.3 (4, 14.9)			10,8 (2.8, 19)	0.3 (2.5, 7.6)	+++
		Rate of improvement FRT^g^, cm, median, IQR	NG		0.4 (0.2, 0.8)			NG	0.0 (0.1, 0.5)	++
		Time Up and Go, sec, median, IQR	34 (23, 48)		11.1 (3.7, 22.1)			29 (21, 48)	8.1 (1.9, 14.2)	–
		6-Minute Walking Test, m, median, IQR	130 (80, 203)		45 (7.5, 91.3)			140 (85, 204)	65 (23.8, 111.2)	–
		Gait velocity, m/s, median, IQR	0.5 (0.3, 0.6)		4.7 (0.7, −10.8)			0.5 (0.3, 0.6)	3 (0.7, 7.8)	–
		Step length, cm, median, IQR	35 (22, 43)		0.2 (0.8, 6)			33 (26, 43)	2 (1.1, 12)	–
		Step test-left, steps, median, IQR	1.5 (0, 6.8)		2 (0, 5.3)			1 (0, 8)	2 (0. 7)	–
		Step test-right, steps, median, IQR	0 (0, 7)		2 (0,5)			2,5 (0, 7.8)	2 (0. 6)	–
		Knee extension – right, kg, median, IQR	10 (7, 16)		2 (0, 4)			11 (9, 15)	1 (−1, 3)	–
		Knee extension – left kg, median, IQR	10 (8, 15)		1 (−1, 3)			12 (9, 16)	0 (−1, 3)	–
		Knee flexion – right kg, median, IQR	8 (6, 11)		1 (0, 4)			8 (6, 11)	1 (−1, 3)	–
		Knee flexion – left kg, median, IQR	8 (6, 10)		2 (0, 4)			8 (6, 10)	0 (−1, 3)	–
		Hip abduction – right kg, median, IQR	6 (4, 8)		1 (0, 3)			6 (5, 8)	1 (−1, 4)	–
		Hip abduction – left kg, median, IQR	5 (3.8, 6)		2 (0, 4)			5 (4, 7)	0.5 (−1, 3.8)	–
		Ankle dorsiflexion – right kg, median, IQR	6 (4, 9)		1 (0, 3)			7 (5, 8.8)	0.5 (−1, 2)	–
		Ankle dorsiflexion – left kg, median, IQR	6 (4,8)		2 (0, 4)			6 (5, 8)	1 (−0.3, 2)	–
Hegerova [22]	2014	**Lean Body Mass, kg**	NG		30.6 (9.1) – pre			NG	30.9 (10.9) – pre	–
		Barthel Index	NG		93.2 (7.7) – pre			NG	91.3 (10.0) – pre	–
Jones [25]	2006	**Modified Barthel Index – median, IQR**	71 (51.5, 83.0)		11 (3.2)			61 (40.5, 82.5)	9 (2.2)	–
		Time Up and Go, decrease in s, median, IQR	24.2 (15.8,37,3)		5.4 (1.0, 12.4)			21.5 (16.9, 25.9)	1.2 (−0.9, 4.3)	+
Kim [15]	2013	**Romberg test (eyes open)**	48.9 (11.6)		−3.7 (17.1)			51.4 (15.3)	−13.0 (18.7)	–
		**Romberg test (eyes closed)**	64.2 (21.0)		−18.1 (26.2)			62.3 (20.3)	−20.4 (23.4)	–
		**Functional Reach Test, cm**	14.8 (5.0)		14.0 (7.4)			15.3 (5.0)	6.9 (7.0)	+
		**Time Up and Go, sec**	18.9 (8.2)		−6.1 (9.3)			20.0 (7.1)	−3.5 (8.4)	+
		**10-Meter Walk Test, sec**	13.5 (6.9)		−6.3 (7.2)			12.4 (5.8)	−2.7 (7.4)	+
Laver [24]	2012	**Time Up and Go, sec**	38.0 (18.8)		−10.1 (33.6)			35.4 (19.1)	−6.6 (22.4)	+
		Modified Berg Balance Scale	28.1 (9.6)		4.0 (11.6)			28.7 (9.8)	1.6 (13.2)	+
		Short Physical Performance Battery	4.0 (2.9)		−0.7 (4.0)			3.4 (2.4)	−0.2 (3.6)	–
		Instrumental Activities of Daily Living Scale, sec	181.0 (110.0)		24.1 (149.1)			141.5 (77.1)	48.1 (120.7)	–
		Functional Independence Measure	100.5 (16.7)		8.2 (23.0)			93.9 (21.3)	14.8 (26.1)	–
		Activities-patient tailored Balance Confidence scale	41.1 (18.0)		0.6 (25.9)			41.8 (20.2)	4.5 (27.7)	–
Maggioni [37]	2009	**Maximal Voluntary Contraction**	NG		KT 3.4 (7.0)	ES 7.8 (5.9)	KT + ES 10.5 (7.3)	NG	−0.3 (6.8)	-*
		**30°/BW quadriceps, N/kg, % of A** **Maximal Voluntary Contraction**	NG		8.2 (6.7)	25.8 (11.3)	16.3 (5.2)	NG	4.9 (5.9)	-*
		**60°/BW quadriceps, N/kg, % of A** **Maximal Voluntary Contraction, finger flexur. N/kg, % of A**	NG		2.7 (3.0)	4.0 (11.1)	7.9 (6.1)	NG	−0.5 (3.0)	-*
		**6-min Walk Test, m, % of A**	NG		14.9 (6.5)	14.0 (4.6)	9.6 (4.3)	NG	8.1 (4.0)	-*
		**Balance, Tinetti test, % of A**	NG		11.3 (4.6)	9.4 (3.7)	11.3 (3.0)	NG	2.0 (4.6)	-*
		**Gait, Tinetti test, % of A**	NG		8.2 (4.2)	9.8 (6.9)	0.8 (3.3)	NG	0.9 (3.2)	-*
de Morton [19]	2007	**Time Up and Go, sec**	35 (30)		−10 (19)			30 (28)	−5 (10)	–
		Barthel Index	66 (26)		12 (16)			68 (26)	10 (14)	–
		Functional Ambulation Categories	4.0 (1.5)		0.7 (1.0)			3.9 (1.6)	0.8 (1.3)	–
Oesch [28]	2017	Objective dynamic balance	1.4 (0.2)		−0.0 (95% CI -0.1, 0.1)			1.4 (0.2)	0.0 (95% CI -0.0, 0.1)	–
Parsons [16]	2016	**Physiological Profile Assessment Score, Quadriceps strength, kg**	28.4 (15.5)		2.6			29.52 (19.1)	2.0	–
		Functional Independence Measure	91.7 (14.6)		12.0			93.2 (13.2)	19.7	+
Raymond [26]	2017	**Elderly Mobility Scale, median, IQR**	11 (7.0, 15)		5			11 (8.0, 15)	5	–
		Berg Balance Scale, median, IQR	30 (20, 4)		8			32 (19, 38)	6	+
		Gait speed (m/s), median, IQR	0.4 (0.3, 0.6)		0.1			0.5 (0.3, 0.6)	0.1	–
		Time Up and Go, sec, median, IQR	29 (20, 42)		−7			29 (23, 42)	−5	–
		Functional Reach Test, cm, median, IQR	8.0 (0, 14)		4			10 (1.5, 15)	4	–
Said [20]	2012	**de Morton Mobility Index**	41.4 (12.9)		9.6 (8.8)			43.2 (16.2)	7.2 (9.2)	–
		Elderly Mobility Scale, median, IQR	15 (7, 17)		NG			NG	12 (7,17)	NG
		Time up and Go, participants	35.5 (11.8)		NG			NG	31.3 (12.4)	NG
		Barthel Index, median, IQR	66 (55, 76)		85.0 (73, 95)			68, (60, 78)	86.5 (68, 98)	–
Tibaek [27]	2013	**Time Up and Go, sec**	25.8 (11.8)		−6 (14.9)			25.7 (14.6)	4.3 (17.7)	–
		**30s-chair stand test, n**	5.0 (3.3)		2.5 (5.2)			4.5 (3.8)	2.3 (4.9)	–
		**10-Meter Walk Test, sec**	19.1 (7.8)		−5.4 (9.1)			18.6 (12.2)	−4.5 (13.1)	–
		Barthel Index transfer	13.1 (3.3)		1.8 (3.4)			13.9 (2.1)	0.3 (3.3)	–
		Barthel Index walking	12.0 (4.5)		2.0 (4.9)			12.4 (3.3)	1.2 (5.2)	–
		Barthel Index stairs	4.2 (4.2)		3.8 (5.4)			5.9 (4.4)	3.3 (4.9)	+
		Modified Functional Ambulatory Categories	11.6 (5.8)		1.6 (9.0)			11.3 (6.8)	3.5 (8.7)	–
Wnuk [18]	2016	**6-min Walk Test, m**	BW 362.3 (41.7)	FW 338.3 (70.8)	BW −39.9 (76.9)	FW −34.0 (101.6)		324.2 (64.0)	−66.2 (88.0)	+**

All variables are presented as mean (SD) unless indicated otherwise. *NG* Not given, *A* Hospital admission, #Primary outcomes in bold, *IQR* Interquartile range, *KT* Kinesiotherapy, *ES* Electrical stimulation, ^a^ = Year of publication, ^b^ = Outcomes included of relevance of this systematic review, ^c^ = Difference between two measurement unless indicated otherwise, ^d^ = Difference between pre and post intervention, ^e^ = Significant difference between intervention and control group: - = *p* > 0.1, +/− = *p* < 0.1 and *p* > 0.05, + = *p* < 0.05, ++ = *p* < 0.01 and +++ = *p* < 0.001, * = no significant difference between all three intervention groups compared with control group, ^f^ = measurement only postoperative, ^g^ = Functional Reach Test, *BW* Backward walking, *FW* Forward walking, ** = significant difference between backward walking group compared with control group

Five of the seven physical interventions that were not continuously adapted to the patient’s capabilities showed positive results on physical performance measures. Additional physical activity by applying therapeutic principles of tai chi had a significant positive effect on fall rates and on balance measured by the Functional Reach Test (FRT), however, no effects were found on outcomes measures for balance, gait ability and muscle strength [23]. Interactive video gaming showed a significantly improved gait ability measured by TUG and balance in the intervention group compared with the control group, but no effect was found on physical performance reflected by Short Physical Performance Battery (SPPB) [24]. An exercise program including exercises for upper limb, lower limb and trunk showed a significant positive effect on functional performance reflected by TUG [25]. Progressive resistance strength training of the lower extremities was found to have a positive effect on balance measured by Berg Balance Scale (BBS) [26] and showed variable effects on functional independence as measured by BI while a significant positive effect on climbing stairs was found and no effect on transfer, walking and physical function [27]. No significant effect of kinesiotherapy or electrical stimulation of the quadriceps or a combination of both was found on balance, gait ability and muscle strength [28] and no effect was found of exergames on balance [29].

## Discussion

Overall, the evidence found for the effect of physical interventions on physical performance in older patients during hospitalization was uncertain. Patient tailored physical interventions were not found to be superior over interventions that were not. Although, the studies were rated of sufficient to good quality, the number of studies and included patients was low and interventions were heterogeneous as well as outcome measures; reporting of intensity of the interventions and adherence were mostly lacking.

This systematic review, focusing on a generic and heterogeneous group of older patients, failed to identify the positive effects of patient tailored physical interventions which is in contrast to studies focusing on specific and more homogenous groups of hospitalized patients like COPD. Interventions like individualized resistance training sessions improved physical performance in hospitalized patients with exacerbation of COPD [30, 31]. Individually tailored modifications to the prescribed intervention are found to contribute to a progressive challenge of the individual capability [32]. Although, next to adaption to the capabilities of the patient, other characteristics of physical interventions like frequency, intensity, duration of the intervention and adherence are critical determinants of its effect.

The frequency of the physical interventions varied widely and seems not to be related with the effect on physical performance in older patients during hospitalization. The physical interventions had a large variance in intensity due to the large variability in intervention types, i.e. progressive resistance training of the lower extremities vs. exercise using interactive video gaming, and in duration of intervention sessions. Previous studies showed that higher intensities result in greater functional improvement in older adults [33–35]. The reason that we were not able to find this may be due to the lack of reported intensities of interventions in the majority of the studies. In a systematic review on the effects of different exercise interventions on functional capacity in physically frail older adults it was concluded that resistance training exercises should include two to three sessions per week, with three sets of 8–12 repetitions at increasing intensity to 80% of one repetition maximum test to improve functional performance [36]. In most studies included in this systematic review the duration of the interventions was determined by the length of hospital stay. Other systematic reviews including physically frail older adults [36] and nursing home residents [33] reported interventions varying between ten weeks to one year and between two to four months respectively. In a systematic review on the effect of interventions with nutrition and exercise in different populations of older adults, a minimum duration of the intervention of three months was suggested to improve physical performance [37]. Considering the short length of hospital stay, the impact of in-hospital physical interventions might be limited by the intervention duration.

This systematic review included a number of studies including a low number of patients, however, the studies were rated of sufficient to good quality. Information on multiple characteristics of the physical interventions were lacking. Therefore, it is inconclusive if the effect of a physical intervention is depended on the intervention being adapted to the capabilities of the patient or that characteristics like frequency, intensity and duration of the physical intervention are more decisive. The question is whether the effects of physical interventions to avoid physical inactivity are sufficient or that more progressive and targeted physical interventions are required. Further research should focus on identifying the dose-effect relationship of both patient tailored and generic physical interventions. This is substantial for hospital policies considering the feasibility of the interventions in clinical practice and the cost efficiency. Physical interventions to avoid physical inactivity are likely to require less resources compared to more progressive and patient tailored interventions.

The outcomes measures used to express physical performance varied widely. As suggested by Cruz-Jentoft et al. (2014) [37], standardization and proper definition of outcome measures for physical performance is needed to compare the effects of physical interventions. None of the studies included an intervention measuring physical activity as an outcome, therefore no evidence was found of the effect of physical interventions on physical activity in older patients during hospitalization.

To the best of our knowledge this the first review focusing on the effect of type of physical interventions on physical performance and physical activity in older patients during hospitalization. It was not possible to perform sub analyses or a quantitative analysis of the data due to the heterogeneity of the interventions and outcomes measures.

## Conclusions

Evidence for the effect of physical interventions on physical performance in older patients during hospitalization was found uncertain. Physical interventions continuously adapted to the capabilities of the patient were not found to be superior compared to interventions that were not. To establish effective interventions, further research is needed on the minimal dose-effect relationship of physical interventions with adequate reporting of frequency, intensity and duration. Meanwhile there is a clear need for standardization and proper definition of outcome measures for physical performance.

## Additional files

Additional file 1: Search syntax. (DOCX 28 kb)

Additional file 2: **Table S1.** Quality assessment of included studies based on PEDro scale. (DOCX 46 kb)

## Abbreviations

10MWT: 10-Meter Walk Test
6MWT: 6-min Walk Test
BBS: Berg Balance Scale
BI: Barthel Index
COPD: Chronic Obstructive Pulmonary Disease
FIM: Functional Independence Measures
FRT: Functional Reach Test
RCTs: Randomized controlled trials
SPPB: Short Physical Performance Battery
TUG: Time Up and Go
