# Supplementary material for: Effect of physical interventions on physical performance and physical activity in older patients during hospitalization: a systematic review
Source: BMC Geriatr. 2018 Nov 23;18:288. doi: 10.1186/s12877-018-0965-2 (PMC6260840; doi:10.1186/s12877-018-0965-2)
Supplement: Supplementary file 1 — Search syntax. (DOCX 28 kb) [file 12877_2018_965_MOESM1_ESM.docx]

**Additional file 1: Search syntax**

| **Database** | **PubMed** |  | **Articles found** |
| --- | --- | --- | --- |
| Date  22-11-2017 | Strategy | Query |  |
| #8 | Add | **(#5 OR #7)** | 690 |
| #7 | Add | **(#4 AND #6)** | 598 |
| #6  (study design) | Add | **((random*[tiab] AND (controlled[tiab] OR control[tiab] OR placebo[tiab] OR versus[tiab] OR vs[tiab] OR group[tiab] OR groups[tiab] OR comparison[tiab] OR compared[tiab] OR arm[tiab] OR arms[tiab] OR crossover[tiab] OR cross-over[tiab]) AND (trial[tiab] OR study[tiab])) OR ((single[tiab] OR double[tiab] OR triple[tiab]) AND (masked[tiab] OR blind*[tiab]))) OR ((random*[ot] AND (controlled[ot] OR control[ot] OR placebo[ot] OR versus[ot] OR vs[ot] OR group[ot] OR groups[ot] OR comparison[ot] OR compared[ot] OR arm[ot] OR arms[ot] OR crossover[ot] OR cross-over[ot]) AND (trial[ot] OR study[ot])) OR ((single[ot] OR double[ot] OR triple[ot]) AND (masked[ot] OR blind*[ot])))** | 606177 |
| #5 | Add | **#3 AND #4** | 3228 |
| #4  (study design) | Add | **#3 AND #4** Filters: **Randomized Controlled Trial** | 571 |
| #3  (intervention) | Add | **"Exercise Movement Techniques"[Mesh] OR "Exercise Therapy"[Mesh] OR exercise[tiab] OR training[tiab]** | 532104 |
| #2  (setting) | Add | **"Hospitalization"[Mesh] OR hospitali*[tiab]** | 341877 |
| #1  (population) | Add | **("Aged"[Mesh] OR "Aged, 80 and over"[Mesh] OR "Frail Elderly"[Mesh] OR "Geriatrics"[Mesh] OR "Geriatric Psychiatry"[Mesh] OR "Geriatric Nursing"[Mesh] OR "Geriatric Dentistry"[Mesh] OR "Dental Care for Aged"[Mesh] OR "Health Services for the Aged"[Mesh]) OR (elder*[tw] OR eldest[tw] OR frail*[tw] OR geriatri*[tw] OR old age*[tw] OR oldest old*[tw] OR senior*[tw] OR senium[tw] OR very old*[tw] OR septuagenarian*[tw] OR octagenarian*[tw] OR octogenarian*[tw] OR nonagenarian*[tw] OR centarian*[tw] OR centenarian*[tw] OR supercentenarian*[tw] OR older people[tw] OR older subject*[tw] OR older patient*[tw] OR older age*[tw] OR older adult*[tw] OR older man[tw] OR older men[tw] OR older male[tw] OR older woman[tw] OR older women[tw] OR older female[tw] OR older population*[tw] OR older person*[tw])** | 2911355 |

| Database | EMBASE |  | Articles found |
| --- | --- | --- | --- |
| Date  22-11-2017 | Strategy | Query |  |
| #6 (study design) | Add | **#3** AND **#4** AND [randomized controlled trial]/lim | 505 |
| #5 | Add | **#3** AND **#4** | 3420 |
| #4 (intervention) | Add | **'kinesiotherapy'**/exp OR **exercise**:ab,ti OR **training**:ab,ti | 697466 |
| #3 | Add | **#1** AND **#2** | 123242 |
| #2 (setting) | Add | **'hospitalization'**/exp OR **hospitali***:ab,ti | 462010 |
| #1 (population) | Add | **'aged'/exp OR 'geriatrics'/exp OR 'elderly care'/exp OR elder*:de,ab,ti OR eldest:de,ab,ti OR frail*:de,ab,ti OR geriatri*:de,ab,ti OR (old NEXT/1 age*):de,ab,ti OR (oldest NEXT/1 old*):de,ab,ti OR senior*:de,ab,ti OR senium:de,ab,ti OR (very NEXT/1 old*):de,ab,ti OR septuagenarian*:de,ab,ti OR octagenarian*:de,ab,ti OR octogenarian*:de,ab,ti OR nonagenarian*:de,ab,ti OR centarian*:de,ab,ti OR centenarian*:de,ab,ti OR supercentenarian*:de,ab,ti OR 'older people':de,ab,ti OR (older NEXT/1 subject*):de,ab,ti OR (older NEXT/1 patient*):de,ab,ti OR (older NEXT/1 age*):de,ab,ti OR (older NEXT/1 adult*):de,ab,ti OR 'older man':de,ab,ti OR 'older men':de,ab,ti OR 'older male':de,ab,ti OR 'older woman':de,ab,ti OR 'older women':de,ab,ti OR 'older female':de,ab,ti OR (older NEXT/1 population*):de,ab,ti OR (older NEXT/1 person*):de,ab,ti** | 2918673 |

| Database | SPORTDiscus |  | Articles found |
| --- | --- | --- | --- |
| Date  22-11-2017 | Strategy | Query |  |
| #7 | Add | **#3** AND **#4** | 26 |
| #6 (study design) | Add | SU RANDOMIZED controlled trials | 7975 |
| #5 | Add | **#3** AND **#4** | 204 |
| #4 (intervention) | Add | DE "**EXERCISE** **therapy**" OR DE "**EXERCISE therapy for older people**" OR TI ( (**exercise** OR **training**) ) OR AB ( (**exercise** OR **training**)) | 222741 |
| #3 | Add | **#1** AND **#2** | 1562 |
| #2 (setting) | Add | DE "**HOSPITAL care**" OR TI **hospitali*** OR AB **hospital*** | 19939 |
| #1 (population) | Add | **( (DE "OLDER people" OR DE "EXERCISE for older people" OR DE "older people -- Recreation" OR DE "PHYSICAL education for older people" OR DE "PHYSICAL fitness for older people" OR DE "sports for older people") OR DE "GERIATRICS” ) OR ( TI (elder* OR eldest OR frail* OR geriatri* OR “old age*” OR “oldest old*” OR senior* OR senium OR “very old*” OR septuagenarian* OR octagenarian* OR octogenarian* OR nonagenarian* OR centarian* OR centenarian* OR supercentenarian* OR “older people” OR “older subject*” OR “older patient*” OR “older age*” OR “older adult*” OR “older man” OR “older men” OR “older male” OR “older woman” OR “older women” OR “older female” OR “older population*” OR “older person*”) OR AB (elder* OR eldest OR frail* OR geriatri* OR “old age*” OR “oldest old*” OR senior* OR senium OR “very old*” OR septuagenarian* OR octagenarian* OR octogenarian* OR nonagenarian* OR centarian* OR centenarian* OR supercentenarian* OR “older people” OR “older subject*” OR “older patient*” OR “older age*” OR “older adult*” OR “older man” OR “older men” OR “older male” OR “older woman” OR “older women” OR “older female” OR “older population*” OR “older person*”) )** | 45841 |

| Database | CINAHL |  | Articles found |
| --- | --- | --- | --- |
| Date  22-11-2017 | Strategy | Query |  |
| #8 | Add | **#6** OR **#7** | 151 |
| #7 (study design) | Add | **#3** AND **#4**  Limiters – Publication Type: Clinical Trial | 142 |
| #6 (study design) | Add | **#3** AND **#4**  Limiters - Clinical Queries: Therapy - High Patient tailoredity | 47 |
| #5 | Add | **#3** AND **#4** | 627 |
| #4 (intervention) | Add | MH "Therapeutic Exercise+" OR TI ( (exercise OR training) ) OR AB ( (exercise OR training) ) | 156092 |
| #3 | Add | **#1** AND **#2** | 18328 |
| #2 (setting) | Add | MH "Hospitalization" OR TI hospitali* OR AB hospitali* | 49897 |
| #1 (population) | Add | **MH "Aged+" OR MH "Aged, 80 and Over" OR MH "Frail Elderly" OR MH "Geriatrics" OR MH "Geriatric Psychiatry" OR MH "Gerontologic Nursing+" OR MH "Gerontologic Care" OR MH "Health Services for the Aged" OR TI (elder* OR eldest OR frail* OR geriatri* OR "old age*" OR "oldest old*" OR senior* OR senium OR "very old*" OR septuagenarian* OR octagenarian* OR octogenarian* OR nonagenarian* OR centarian* OR centenarian* OR supercentenarian* OR "older people" OR "older subject*" OR "older patient*" OR "older age*" OR "older adult*" OR "older man" OR "older men" OR "older male" OR "older woman" OR "older women" OR "older female" OR "older population*" OR "older person*") OR AB (elder* OR eldest OR frail* OR geriatri* OR "old age*" OR "oldest old*" OR senior* OR senium OR "very old*" OR septuagenarian* OR octagenarian* OR octogenarian* OR nonagenarian* OR centarian* OR centenarian* OR supercentenarian* OR "older people" OR "older subject*" OR "older patient*" OR "older age*" OR "older adult*" OR "older man" OR "older men" OR "older male" OR "older woman" OR "older women" OR "older female" OR "older population*" OR "older person*")** | 461092 |

| Database | Cochrane libary |  | Articles found |
| --- | --- | --- | --- |
| Date  26-10-2015 | Strategy | Query |  |
| #5 | Add | **#3** AND **#4 in Trials** | 273 |
| #4 (intervention) | Add | (exercise or training):ti,ab,kw in Trials | 83942 |
| #3 | Add | **#1** AND **#2 in Trials** | 2534 |
| #2 (setting) | Add | hospitali*:ti,ab,kw in Trials | 29484 |
| #1 (population) | Add | **(elder* or eldest or frail* or geriatri* or "old age*" or "oldest old*" or senior* or senium or "very old*" or septuagenarian* or octagenarian* or octogenarian* or nonagenarian* or centarian* or centenarian* or supercentenarian* or "older people" or "older subject*" or "older patient*" or "older age*" or "older adult*" or "older man" or "older men" or "older male" or "older woman" or "older women" or "older female" or "older population*" or "older person*"):ti,ab,kw in Trials** | 40388 |
