# Supplementary material for: Effect of physical interventions on physical performance and physical activity in older patients during hospitalization: a systematic review
Source: BMC Geriatr. 2018 Nov 23;18:288. doi: 10.1186/s12877-018-0965-2 (PMC6260840; doi:10.1186/s12877-018-0965-2)
Supplement: Supplementary file 2 — Table S1. Quality assessment of included studies based on PEDro scale. (DOCX 46 kb) [file 12877_2018_965_MOESM2_ESM.docx]

Table S1: Quality assessment of included studies based on PEDro scale.

| **Reference** | **Criteria** | | | | | | | | | | | **Total** | **Quality** |
| --- | --- | --- | --- | --- | --- | --- | --- | --- | --- | --- | --- | --- | --- |
|  | **1*** | **2** | **3** | **4** | **5** | **6** | **7** | **8** | **9** | **10** | **11** |  |  |
| Bürge, 2017 [21] | 1 | 1 | 0 | 1 | 0 | 1 | 0 | 0 | 0 | 1 | 1 | 5 | Sufficient |
| Czyzewski, 2013 [17] | 1 | 1 | 0 | 1 | 0 | 0 | 0 | 0 | 0 | 1 | 1 | 4 | Sufficient |
| Haines, 2007 [23] | 1 | 1 | 1 | 1 | 0 | 0 | 1 | 0 | 1 | 1 | 1 | 7 | Good |
| Hegerova, 2014 [22] | 1 | 1 | 0 | 1 | 0 | 0 | 0 | 1 | 1 | 1 | 1 | 6 | Good |
| Jones, 2006 [25] | 1 | 1 | 0 | 0 | 0 | 0 | 1 | 0 | 1 | 1 | 1 | 5 | Sufficient |
| Kim, 2013 [15] | 1 | 1 | 0 | 1 | 0 | 0 | 0 | 1 | 1 | 1 | 1 | 6 | Good |
| Laver, 2012 [24] | 1 | 1 | 1 | 1 | 0 | 0 | 1 | 1 | 1 | 1 | 1 | 8 | Good |
| Maggioni, 2009 [28] | 1 | 1 | 1 | 1 | 0 | 0 | 1 | 1 | 1 | 1 | 1 | 8 | Good |
| De Morton, 2007 [19] | 1 | 1 | 1 | 1 | 1 | 0 | 0 | 1 | 1 | 1 | 1 | 8 | Good |
| Oesch, 2017 [29] | 1 | 1 | 1 | 1 | 0 | 0 | 0 | 1 | 1 | 1 | 1 | 7 | Good |
| Parsons, 2016 [16] | 1 | 1 | 1 | 1 | 0 | 0 | 0 | 1 | 1 | 1 | 1 | 7 | Good |
| Raymond, 2017 [26] | 1 | 1 | 1 | 1 | 0 | 0 | 1 | 1 | 1 | 1 | 1 | 8 | Good |
| Said, 2012 [20] | 1 | 1 | 1 | 1 | 0 | 0 | 1 | 1 | 1 | 1 | 1 | 8 | Good |
| Tibaek, 2013 [27] | 1 | 1 | 0 | 1 | 0 | 0 | 1 | 0 | 1 | 1 | 1 | 6 | Good |
| Wnuk, 2016 [18] | 1 | 1 | 0 | 1 | 0 | 0 | 1 | 1 | 1 | 1 | 1 | 7 | Good |

Column numbers correspond the following criteria on the PEDro scale: 1= Eligibility criteria were specified, 2= Participants were randomly allocated to groups, 3= Allocation was concealed, 4= Groups were similar at baseline, 5= Participants were blinded, 6= Therapists were blinded, 7= Assessors were blinded, 8= Measures of at least one key outcome were obtained from >85% of the participants, 9= Intention-to-treat analysis was performed for at least one key outcome, 10= Between-group statistical comparison was reported for at least one key outcome, 11= Point estimates and variability measures were provided for at least one key outcome. 1= Criterion was satisfied, 0= Criterion was not satisfied, *criterion not included in total score. Quality: 0-3= Insufficient, 4-5= Sufficient, 6-8= Good, 9-10= Excellent.
